# Dosimetric benefit of an adaptive treatment by means of plan selection for rectal cancer patients in both short and long course radiation therapy

**DOI:** 10.1186/s13014-020-1461-3

**Published:** 2020-01-13

**Authors:** R. de Jong, J. Visser, K. F. Crama, N. van Wieringen, J. Wiersma, E. D. Geijsen, A. Bel

**Affiliations:** Department of Radiation Oncology, Amsterdam UMC, University of Amsterdam, Meibergdreef 9, 1105AZ Amsterdam, The Netherlands

**Keywords:** Adaptive radiotherapy, Adaptive treatment, Rectal cancer, Plan selection, Library of plans, Plan of the day, Normal tissue sparing

## Abstract

**Background:**

To compare target coverage and dose to the organs at risk in two approaches to rectal cancer: a clinically implemented adaptive radiotherapy (ART) strategy using plan selection, and a non-adaptive (non-ART) strategy.

**Methods:**

The inclusion of the first 20 patients receiving adaptive radiotherapy produced 10 patients with a long treatment schedule (25x2Gy) and 10 patients with a short schedule (5X5Gy). We prepared a library of three plans with different anterior PTV margins to the upper mesorectum, and selected the most appropriate plan on daily Conebeam CT scans (CBCT). We also created a non-adaptive treatment plan with a 20 mm margin. Bowel bag, bladder and target volume were delineated on CBCT. Daily DHVs were calculated based on the dose distribution of the selected and non-adaptive plans. Coverage of the target volume was compared per fraction between the ART and non-ART plans, as was the dose to the bladder and small bowel, assessing the following dose levels: V15Gy, V30Gy, V40Gy, V15Gy and V95% for long treatment schedules, and V15Gy and V95% for short ones.

**Results:**

Target volume coverage was maintained from 98.3% (non-ART) to 99.0% (ART)(*p* = 0.878). In the small bowel, ART appeared to have produced significant reductions in the long treatment schedule at V15Gy, V40Gy, V45Gy and V95% (*p* <  0.05), but with small absolute differences. The DVH parameters tested for the short treatment schedule did not differ significantly. In the bladder, all DVH parameters in both schedules showed significant reductions (*p* <  0.05), also with small absolute differences.

**Conclusions:**

The adaptive treatment maintained target coverage and reduced dose to the organs at risk.

**Trial registration:**

Medical Research Involving Human Subjects Act (WMO) does not apply to this study and was retrospectively approved by the Medical Ethics review Committee of the Academic Medical Center, W19_194 # 19.233.

## Background

Due to the inevitable dose to organs at risk (OAR) such as the small bowel and bladder, radiation therapy for rectum cancer is associated with toxicity [1]. While treatment-planning techniques with intensity modulation (IMRT/VMAT) make it possible to reduce the dose to OARs by steep dose gradients, the benefit is counteracted by the large population-based margins that are necessary to compensate for large inter-fraction shape-changes caused by changing rectum and bladder filling [2–6]. Drinking protocols to stabilize the volume of the bladder have had only limited success [7, 8]. Because the digestive system is both complex and deregulated by a tumor [9], there are also no clear instruments for stabilizing rectal volume. Although a diet (i.e. directions on fluid and fiber intake) or laxatives may help [7, 10–12], they burden the patient. Nijkamp et al [3, 4, 6] report geometrical uncertainties of the mesorectum that, in rectal cancer, require population-based margins up to 24 mm.

To cope with inter-fraction shape changes in cervix and bladder cancer patients several groups introduced adaptive strategies with plan selection [13–17]. This entails creating multiple plans tailored to possible shapes and for these two sites the shape of the target volume can largely be predicted by acquiring two planning CT scans capturing the extreme bladder fillings (full and empty bladder). Structures of the target volume based on these two CT scans can be interpolated to generate intermediate structures (or even extrapolated if necessary) for treatment planning. For each of these plans smaller margins than used for non-ART will suffice. Subsequently, the best fitting plan will be selected based on daily CBCT [18].

For rectal cancer patients, the shape-changes in the target volume are driven mainly by the rectal volume [3, 4] and for that reason creating multiple based on varying bladder filling is not useful. Therefor we developed plan selection based on *variable margins* to the upper anterior side of the mesorectum, which is the part of the target volume with the largest deformations. The remaining part of the upper mesorectum is enclosed by bony anatomy (dorsal) or the elective lymph node region (lateral) and for that reason not eligible for variable margins. These multiple PTV margins were based on a single planning CT scan with spontaneous rectum filling. For implementation purposes, our group has already simulated this strategy for its potential dosimetric effect [19] and also to test the feasibility of selecting a margin based on CBCT images [20]. So far this strategy has not been evaluated within a clinical setting for long-course (LCRT) and short-course radiotherapy treatment (SCRT) in which patients are treated in a supine-only position.

We therefore compared target coverage and dose to the organs at risk in two approaches to rectal cancer: a clinically implemented adaptive radiotherapy (ART) strategy using plan selection, and a non-adaptive (non-ART) strategy.

## Methods

In this study we used the same methodology as that used in our previous study [19], but applied to a clinical cohort of LCRT (25x2Gy) and SCRT (5x5Gy).

### Patients

We included 20 patients, who were treated consecutively between May and August 2016. LCRT and SCRT were both eligible for plan selection. This resulted in the inclusion of 10 LCRT patients and 10 SCRT patients, with a total of 300 CBCT scans. Patient details are shown in Table 1.

**Table 1:** Patient characteristics

nr	Age	Sex	Tumor stage	Treatment scheme	Chemo	GTV location	Surgery	Bladder volume cm^3^ planning CT	Upper mesorectum volume cm^3^ planning CT	Upper mesorectum length mm planning CT	Available margins (mm)			Average bladder volume CBCT relative to planning CT	Average upper mesorectum volume CBCT relative to planning CT
1	65	M	cT3N1M0	5 × 5 Gy	N	proximal	LAR	185	259	55	−15	0	15	1.13	1.03
2	70	F	cT3N0M0	5 × 5 Gy	N	mid	LAR	558	143	60	0	15	25	0.66	1.05
3	63	M	cT4N1M0	5 × 5 Gy	Y	distal	ns	203	269	60	−15	0	15	0.84	1.06
4	71	M	cT3N1M0	5 × 5 Gy	N	mid	LAR	622	184	86	0	15	25	0.70	1.14
5	74	M	cT3N0M0	5 × 5 Gy	N	proximal	LAR	557	226	65	0	15	25	0.57	1.06
6	56	F	cT3N1M0	5 × 5 Gy	N	mid	LAR	135	156	41	−15	0	15	0.42	1.02
7	73	M	cT3N2M1	5 × 5 Gy	Y	proximal	LAR	160	357	71	0	15	25	1.02	0.97
8	70	M	cT3N2M0	5 × 5 Gy	N	distal	APR	295	244	65	0	15	25	0.59	1.03
9	40	F	cT3N1M0	5 × 5 Gy	N	mid	LAR	941	131	57	0	15	25	0.52	1.25
10	78	M	cT4N1M0	5 × 5 Gy	N	rectosigmoid	LAR	613	289	70	0	15	25	0.33	1.06
11	71	F	cT3N1M0	25 × 2 Gy	Y	proximal	LAR	791	393	80	−15	0	15	0.63	0.71
12	66	M	cT3N2M0	25 × 2 Gy	Y	mid	LAR	150	185	46	−15	0	15	1.05	0.9
13	64	F	cT3N2M0	25 × 2 Gy	Y	distal	APR	403	223	65	0	15	25	0.31	0.95
14	65	M	cT3N2M0	25 × 2 Gy	Y	proximal	LAR	446	309	65	0	15	25	0.65	1.09
15	55	F	cT4N0M0	25 × 2 Gy	Y	distal	LAR	595	186	67	0	15	25	0.42	0.91
16	59	M	cT3N0M0	25 × 2 Gy	Y	mid	LAR	166	170	52	−15	0	15	1.27	1.00
17	72	M	cT3N2M0	25 × 2 Gy	Y	proximal	LAR	420	373	85	0	15	25	0.65	1.05
18	69	F	cT4N1M0	25 × 2 Gy	Y	mid	LAR	511	442	97	0	15	25	0.79	0.99
19	77	M	cT3N2M0	25 × 2 Gy	Y	distal	ns	456	128	58	0	15	25	0.55	1.10
20	60	M	cT3N2M0	25 × 2 Gy	Y	distal	ns	392	261	67	−15	0	15	0.75	0.92

*ns* Not specified

*LAR* Low anterior resection

*APR* Abdominal perineal resection

The upper mesorectum lies between the presacral space and lower mesorectum. As these each have a 1 cm caudal and cranial margin, we made a pragmatic decision only to include patients for plan selection if the length of the upper mesorectum (measured from the base of the bladder) was over 4.5 cm. This would leave at least 2.5 cm for variable margins to the ventral side of the upper mesorectum. Patients were positioned supine with knee support and a device to position the arms above the head (Posirest, CIVCO).

### Planning CT and delineations

A planning CT scan was acquired with a full bladder, instructions having been to empty the bladder 1.5 h before scanning and then to drink 0.5 l of water. As no instructions had been given with regard to rectal filling, spontaneous rectum filling was used.

**Fig. 1:**
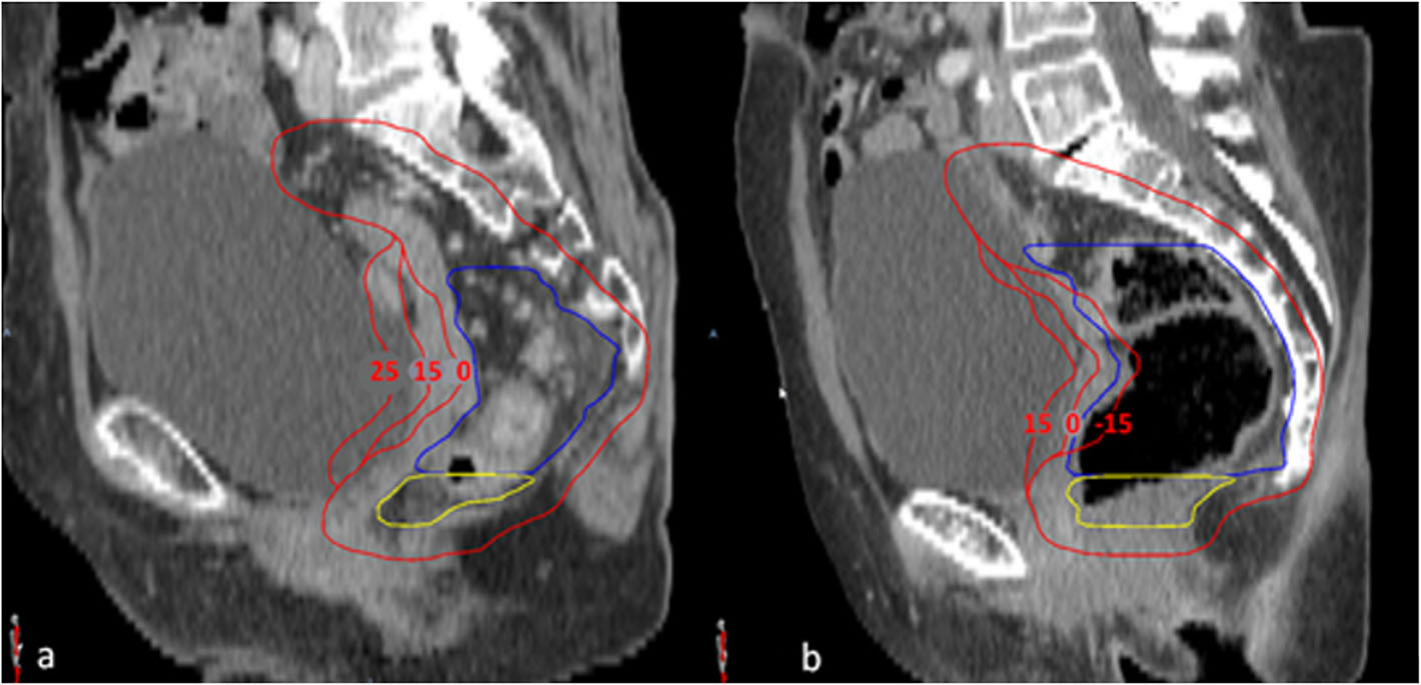
Margin sets based on anatomy as captured on planning CT. **a** shows an empty rectum with a set of 25 mm, 15 mm, and 0 mm margins (red) for the upper mesorectum (blue). **b** shows a full rectum with a set of 15 mm, 0 mm, and − 15 mm anterior margins (red) for the upper mesorectum (blue). Yellow is the lower mesorectum

For GTV, the gross tumor volume and pathologic lymph nodes were delineated. For CTV the mesorectum, presacral space, internal iliac lymph node regions and, when applicable, obturator lymph node regions, were delineated by a radiation oncologist according to the guidelines by Roels et al. [21] (Advantage SIM, GE or VelocityAI 3.2, Varian Medical Systems). To be able to differentiate margins between the upper and lower mesorectum based on the geometrical uncertainties reported by Nijkamp et al [3, 4, 6], the mesorectum was divided into an upper and lower part, with the transition at the base of the bladder (Fig. 1). Total CTV volume was created by combining all CTV regions. Radiation therapists (RTTs) contoured the OARs (i.e., the bladder, bowel bag and femur heads) according to RTOG guidelines [22].

### Treatment planning

Planning CT and delineations were imported into the treatment-planning system (Oncentra 4.5, Elekta AB, Sweden). PTV margins were created (VelocityAI) by expanding the lymph-node regions by 8 mm and the presacral space by 10 mm. The upper and lower mesorectum were expanded in all directions by 10 mm, except for the anterior side. The anterior side of the lower mesorectum was expanded by 15 mm. The anterior margin to the upper mesorectum was variable. To simplify the plan-selection process, we chose 15 mm as the difference between the PTV margins, except for the largest PTV margin, for which – on the basis of the maximum uncertainty found by Nijkamp – we chose 25 mm.

To reduce the number of PTVs in order to minimize workload at treatment planning, two sets of margins were defined, according to the anatomy captured on the planning CT scan: If a rectum was deemed empty after visual inspection on planning CT we used PTV margins of 25 mm, 15 mm, 0 mm, as − 15 mm was unlikely to be needed. Conversely, if a rectum was deemed full after visual inspection on planning CT, we used 15 mm, 0 mm and − 15 mm, as 25 mm was unlikely to be needed. Per patient, this resulted in 3 PTV margins, and thus 3 plans from which we could select during treatment (Fig. 1).

To compare this adaptive treatment with the former non-adaptive strategy, we generated an extra treatment plan in which all margins were kept the same, but in which a fixed anterior margin of 20 mm to the anterior upper mesorectum was used rather than a variable margin. Previously, before the implementation of the plan-selection strategy, this margin was the standard of care. Patients were planned with a 10 MV dual-arc VMAT technique. All treatment plans were checked for clinical acceptability by an experienced RTT and a medical physicist.

### Plan selection

Conebeam CT (CBCT) scans were registered on pelvic bony anatomy (XVI5.0, Elekta) including translations and rotations with a maximum tolerance on rotations of 4 degrees. If set-up exceeded rotational tolerance, a patient was re-aligned. The registration results including rotations were converted into a correction with translations-only by taking out the rotations using a rotation point at the center of gravity of the PTV.

This resulted in a table translation, which was then applied. At the treatment machine, trained RTTs selected the smallest PTV that encompassed the complete clinical target volume on daily CBCT scans [20]. Retrospectively, the selected margins were reviewed by a single expert to check concordance with the clinical guidelines.

### Dose calculation and comparison

Each CBCT scan was exported to VelocityAI. The patient’s position on this CBCT scan is as it was during irradiation, i.e. translational errors were corrected using an online position verification protocol. Rotational errors are still present, as these cannot be corrected using our treatment couch. On each CBCT, a single observer delineated the upper and lower mesorectum based on the original clinical delineations of the radiation oncologist, as well as the bladder and bowel bag for the small bowel. Using identity transformation, delineations were propagated to the planning CT scan. The dose distribution planned was used to calculate daily DVHs for the delineations propagated, both for the selected treatment plan and for the fixed margin plan (20 mm)(version R2015b, MathWorks, Natick). Since the dose was not recalculated, we disregarded changes in dose distribution that resulted from changing anatomy.

### Statistical analysis

Descriptive statistics were used to describe the distribution of plan selection for the total cohort and per individual patient.

To test the correlation of rectum volume with the selected plan, we calculated volumes relative to the planning CT scan of the upper mesorectum on daily CBCT. Because 6 combinations of different margins were tested, we used Bonferroni correction for multiple comparison testing after one-way ANOVA resulting in a confidence level of 0.05/6 = 0.008.

Using Wilcoxon signed-rank the difference in coverage between ART and non-ART was tested *per fraction* for the combined upper and lower mesorectum. Coverage was expressed as V95%, the volume receiving at least 95% of the prescribed dose.

Because deformable registration was not considered accurate enough [23], dose accumulation was not used to assess dose to OARs. For this reason, the difference of the dose to the OARs between the ART and non-ART strategy had to be tested per fraction. Because the literature on predictive dose volume parameters is relatively sparse we used a range of DVH parameters based on the parameters suggested in the QUANTEC papers [24, 25] and the DVH parameters suggested by Moutet-Audoard et al. [26] and Devill et al. [27, 28] (i.e., the volume receiving at least 15Gy (V15Gy), 30Gy (V30Gy), 40Gy (V40Gy), 45Gy (V45Gy)) per fraction. For LCRT, these were the following: 1.) the volumes that received at least 0.6Gy (V0.6Gy); 1.2Gy (V1.2Gy); 1.6Gy (V1.6Gy); and 1.8Gy (V1.8Gy) per fraction; 2.) the volume that received at least 95% of the prescribed dose (V95%); and 3.) the mean dose (Dmean) for bladder. For SCRT, 15Gy equals 3.0Gy per fraction; other dose levels are higher than the dose prescribed for SCRT. The V95% for LCRT is therefore the only dose level we evaluated together with the V95% for SCRT. We also tested Dmean for the bladder. All dose levels were tested for significant differences using the Wilcoxon signed-rank test.

Significance for coverage and dose to the OARs was set at *p* <  0.05. Statistical analysis was performed using SPSS24.

## Results

### Patients and plan selection

**Fig. 2:**
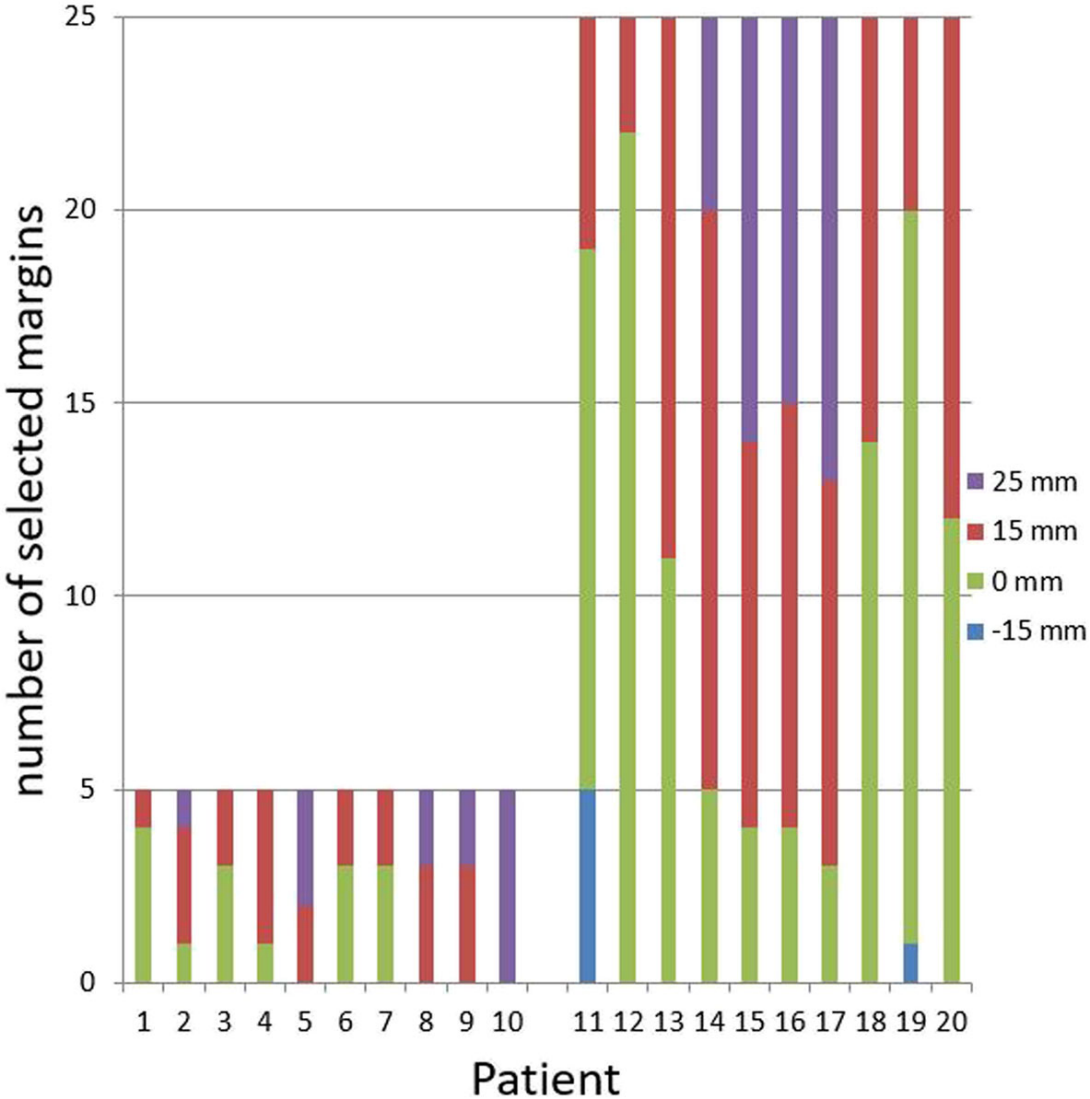
Distribution of selected margins per patient sorted on short (5 × 5Gy) and long (25x2Gy) treatment schedules

For clinical adaptive treatment, the margin set of 25 mm, 15 mm and 0 mm was used for 13 patients, and the margin set of 15 mm, 0 mm, and − 15 mm was used for 7 patients. Overall, based on daily CBCT scans, the − 15 mm margin was selected in 2% of fractions, the 0 mm margin was selected in 41%, the 15 mm margin in 40%, and the 25 mm margin in 17%. The distribution of selected margins per patients is shown in Fig. 2. For one patient only, one specific plan (25 mm margin) was used for all 5 fractions. All available plans were used for 7 patients.

**Fig. 3:**
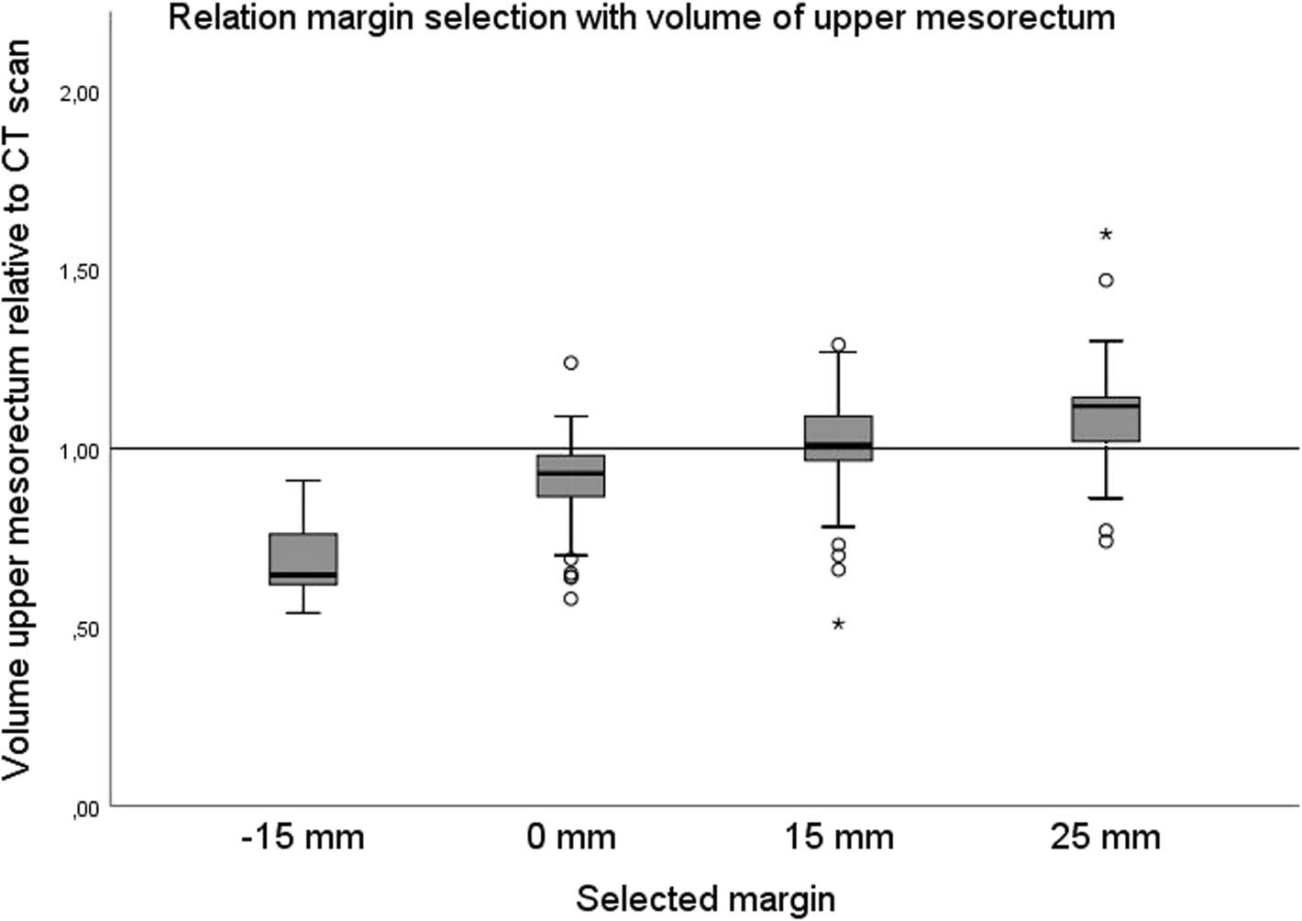
Boxplot shows the relationship between the upper mesorectum volume on CBCT relative to the planning CT scan with selected margin. It shows the interquartile range, with a horizontal line showing the group median. Whiskers indicate the 5th and 95th percentiles. Outliers are marked. One-way ANOVA testing with Bonferroni correction applied showed all margins to be significantly different (*p* <  0.001)

For each margin selected, the relative volume of the mesorectum differed significantly from the relative volume of the mesorectum of the other margins (*p* <  0.001). The graph shows that an increase in the selected margin was accompanied by an increase in relative volume (Fig. 3).

Our retrospective review of concordance with clinical selection guidelines showed that a smaller PTV could have been selected for 20% of fractions and a larger PTV should have been selected for 2%.

### Target coverage

The average percentage of the mesorectum receiving at least 95% of the prescribed dose increased from 98.3 to 99.0%, for all patients and all fractions. This was not statistically significant (*p* = 0.878).

### Dose to the organs at risk

**Fig. 4:**
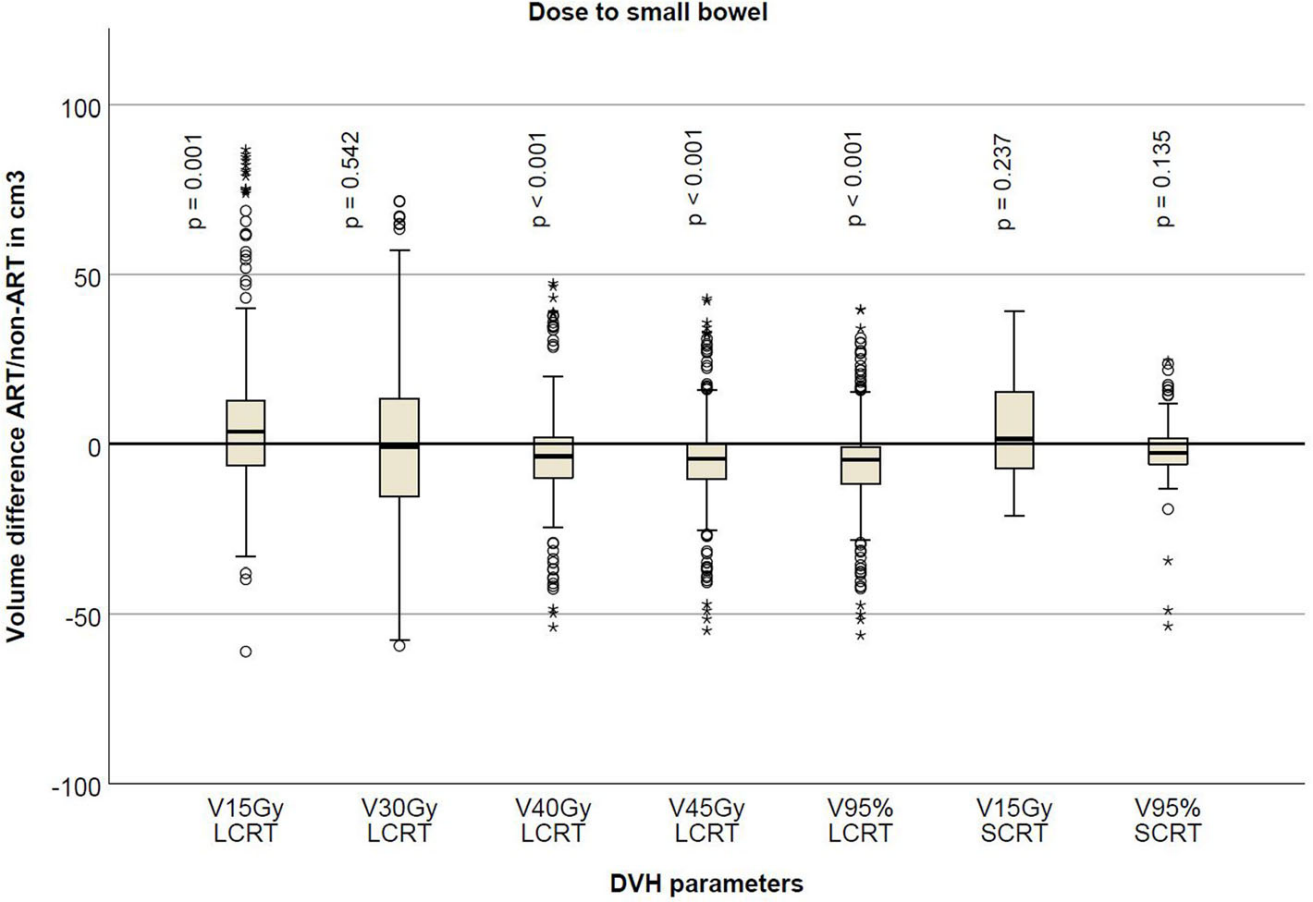
Boxplot showing difference in volume in cm3 for the small bowel for the different DVH parameters tested for long- and short-course radiation therapy. Negative volume favors the plan-selection strategy. The boxplot shows the interquartile range. Whiskers indicate the 5th and 95th percentiles. Outliers with values between 1.5 and 3.0 IQR (open circle) and extremes > 3.0 IQR (asterisk) are marked

The adaptive treatment for LCRT significantly reduced small bowel V15Gy, V40Gy, V45Gy and V95%, the average volume reduction being approximately 8 cm3. V15Gy and V95% for SCRT were not significantly different (Table 2, Fig. 4).

**Table 2:** Dose to the organs at risk for all patients and all fractions

	Dose per fraction		Median values (range)		*p*-value
			ART	Non-ART	
LCRT (25 × 2 Gy)					
Bladder	V15Gy	(%)	99.8 (87.6–100.0)	100.0 (85.6–100.0)	< 0.001
	V30Gy	(%)	43.0 (12.6–99.3)	49.6 (19.1–96.8)	< 0.001
	V40Gy	(%)	22.8 (1.8–91.1)	29.0 (4.2–85.3)	< 0.001
	V45Gy	(%)	15.0 (0.2–87.0)	21.8 (0.4–77.6)	< 0.001
	V95%	(%)	10.8 (0.0–82.2)	17.8 (0.0–72.4)	< 0.001
	Dmean	(Gy)	1.2 (0.9–2.0)	1.3 (1.0–1.9)	< 0.001
Small Bowel	V15Gy	(cm3)	847 (332–1447)	853 (294–1363)	0.001
	V30Gy	(cm3)	309 (119–554)	309 (120–557)	0.542
	V40Gy	(cm3)	205 (99–431)	214 (99–434)	< 0.001
	V45Gy	(cm3)	179 (93–381)	187 (90–390)	< 0.001
	V95%	(cm3)	160 (83–358)	170 (82–366)	< 0.001
SCRT (5 × 5 Gy)					
Bladder	V15Gy	(%)	29.7 (5.7–58.6)	33.8 (9.9–60.5)	0.001
	V95%	(%)	4.4 (0.0–16.4)	7.1 (0.0–27.7)	0.013
	Dmean	(Gy)	2.7 (2.1–3.5)	2.8 (2.3–3.6)	0.001
Small Bowel	V15Gy	(cm3)	329 (139–456)	317 (137–477)	0.237
	V95%	(cm3)	176 (87–274)	191 (93–275)	0.135

Total dose V15Gy, V30Gy, V40Gy, V45Gy for LCRT corresponding to fraction dose V0.6Gy, V1.2Gy, V1.6Gy, V1.8Gy respectively and V15Gy total dose for SCRT corresponding to V3.0Gy fraction dose

**Fig. 5:**
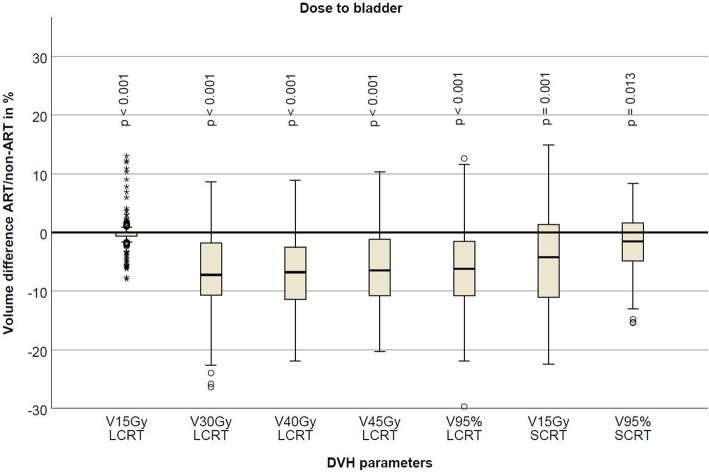
Boxplot showing difference in volume in percentage for bladder for the different DVH parameters tested for long and short-course radiation therapy. Negative volume favors the plan-selection strategy. The boxplot shows the interquartile range (IQR). Whiskers indicate the 5th and 95th percentiles. Outliers with values between 1.5 and 3.0 IQR (open circle) and extremes > 3.0 IQR (asterisk) are marked

For both treatment schemes, the adaptive treatment significantly reduced all dose volume parameters in the bladder. The difference for V15Gy is very small but the average percentage reduction is approximately 7% (Table 2, Fig. 5).

In a subset of patients, the benefits were greater. In the bladder, for example, patient 7 (SCRT) had maximum average differences of up to 15% for V15Gy and of up to 12% for V95%. In the bowel, this patient had maximum average differences of up to12cm3 for V15Gy and of up to 35 cm3 for V95%. Similarly, for the bladder, patient 18 (LCRT) had maximum average differences of up to 11% for both V45Gy and V95%; and of up to 21 cm3 for both V45Gy and V95% for small bowel.

## Discussion

This paper provides the first dosimetric comparison between a clinically implemented adaptive treatment and a non-adaptive treatment in external radiation therapy for rectal cancer. Based on a single CT scan, the plan-selection strategy used variable anterior margins to the upper mesorectum, and the margin was selected based on daily CBCT. This adaptive treatment maintained coverage of the target volume and reduced the dose to the small bowel and bladder.

The majority of the tested dose levels were significantly better but the absolute differences for the total cohort were small. However, for individual patients there can be substantial benefits and this raises the question about costs and potential benefit. For our department, where daily online CBCT imaging is standard and plan selection is also used for cervix and bladder, implementing plan selection for rectum was rather straightforward. Procedures, education and modifications of the technical infrastructure could be reused from the earlier implementations. However, plan adaptation for rectum may not be the first tumor type of choice when starting with a plan selection procedure from scratch.

A limitation of our study is the rather small sample size. Coincidentally, LCRT and SCRT were of equal size in this clinical cohort. Due to their different fraction doses, the two treatment schemes were analyzed separately. While Nijkamp et al. [3, 4, 6] describe different geometrical uncertainties for LCRT and SCRT, and also for male and female, prone and supine, the sample size in our cohort was too small to compare the two treatment schemes with respect to the benefit of plan selection. This cohort of 20 patients with a total of 300 fractions is sufficient to test the difference between ART and non-ART because data of different fractions within one patient can be considered independent due to large day-to-day variation of the mesorectum and OARs. The different DVH parameters that were considered, are not expected to be independent, but, because literature on IMRT/VMAT based dose volume predictors is sparse and inconclusive, we reported all tested DVH parameters anyway.

In this cohort, the dose to the small bowel (V15Gy, V95%) was not found to be significantly different for SCRT between ART and non-ART, whereas it was significantly different for LCRT (V15Gy, V40Gy, V45Gy and V95%). This difference may have been due to the time trend towards smaller rectum volumes described in the literature for long-course treatments in the prostate and rectum [6, 29]. It may also have been caused by the limited sample size of 10 patients, each of whom received only 5 fractions.

A second limitation of our study is the comparison of the dose levels per fraction. The evaluation of dose levels per fraction was based on the corresponding total dose levels, such as V0.6Gy for V15Gy (LCRT). Evaluating the actual total dose for the entire treatment would require the accumulation of the fractional dose distribution, for which the deformable image registration algorithms available are not sufficiently accurate [23]. This explains our decision to test the difference doses to OARs between the adaptive and non-adaptive strategy per fraction.

In this study the initially planned dose in combination with the structures as delineated on CBCT were used to evaluate coverage of the target volume and dose to the OARs, because dose calculation based solely on CBCT scans has uncertainties since CBCT grey values were not calibrated. As a consequence, the dosimetric effect of anatomical changes (for example, air in rectum) was not taken into account. Alternatively, the planning CT could be deformably registered to the CBCT to use for dose calculation. Deformable image registration, especially in the presence or absence of air, has its limitations as well. Independent of the method used for recalculation of the dose, anatomical changes would affect the results for both ART and non-ART to some extent and not so much the difference between ART and non-ART.

**Fig. 6:**
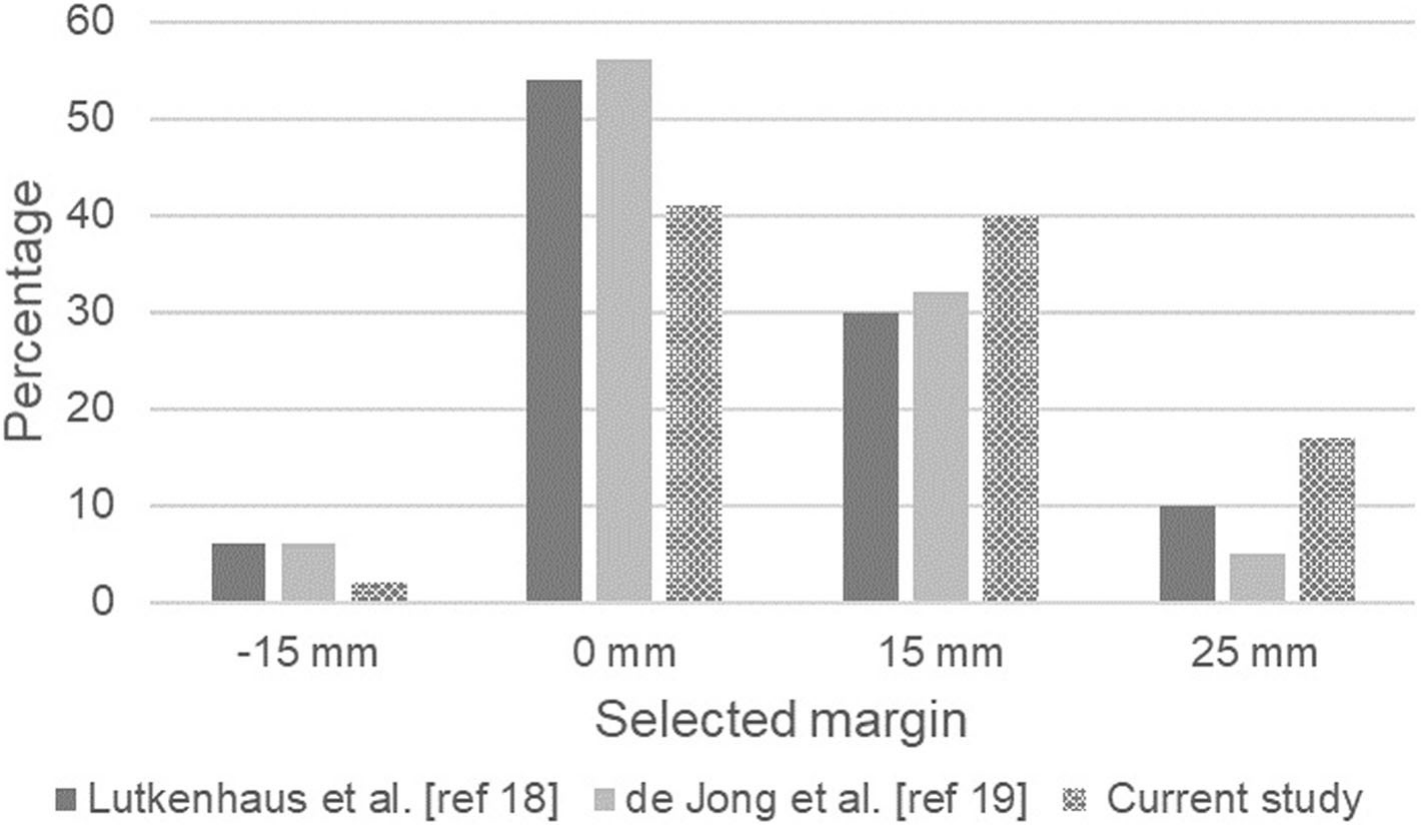
Bar chart showing the distribution of selected margins. Solid grays show two comparable retrospective studies; the dotted bar shows the current clinical study. The chart shows a shift towards larger plans under clinical conditions

Our results are similar to those in our study (see Lutkenhaus et al.) [19], which, in SCRT only, describes the dosimetric benefit of plan selection in a simulation planning study conducted as part of our implementation strategy. Our current prospective study shows that the dosimetric benefit of the adaptive treatment remains in a clinical setting. What did change was the distribution of plans. In approximate terms, while selection of the 15 mm plan increased from approximately 30 to 40%, and selection of the 25 mm plan increased from 8 to 17%, selection of the 0 mm plan fell from 55 to 41% (Fig. 6) [19, 20]. As observers in a simulated study have more time to evaluate images and make hypothetical decisions than in clinical decision-making involving an actual patient, this may have resulted in a change of the distribution towards larger plans with more certainty about the target coverage in cases involving challenging image quality. This would be consistent with the retrospective review, which showed that a smaller plan could have been selected in 20% of fractions and a larger margin should have been selected in 2% of fractions. Even with this shift towards larger plans, the benefit of plan selection remains. Improving CBCT image quality might increase confidence, and also increase the benefits of plan selection.

In this study, the average extent to which the OARs were spared was limited. The re-planning strategy proposed by Nijkamp et al. [30] delivers more sparing to the OARs, as it does more than merely compensate the variability of the upper mesorectum. This strategy is based on 5 repeat CT scans and 5 repeat delineations followed by an new plan of an updated CTV structure, which deliver a 34 cm3 reduction to the bowel area for V15Gy, and a 30 cm3 reduction for V45Gy. Our study reports median reductions from 853 cm3 to 847 cm3 and 187 cm3 to 179 cm3 V15Gy and V45Gy. For the bladder, Nijkamp et al. reported a reduction in mean dose to the bladder of 2.5Gy, compared to the median reduction of 2.8Gy to 2.7Gy we found in our study. While Nijkamp’s initial anterior PTV margin to the upper mesorectum was 24 mm, our strategy compares to a 20 mm anterior PTV margin. The approach proposed by Nijkamp has a higher workload than the strategy proposed in this study, which is based on a single CT scan and single delineation. The extra workload in our adaptive strategy is incurred at treatment planning, thus adding 120 min to the total workflow.

Passoni et al. and Raso et al [31, 32] also reported on an adaptive procedure, but applied to the boost of the residual tumor during the last 6 fractions of LCRT. Byskov et al. [33] describe an adaptive approach to re-irradiation of rectal recurrence. As neither strategy is applied to the mesorectum, they cannot easily be compared.

A practical hurdle to the widespread adoption of plan selection is that if, in current commercial systems, a margin is selected that best fits the target on the CBCT, the corresponding plan in the delivery system has to be selected manually. Software support for an automatic plan delivery after selection of the plan would make the procedure less error-prone.

To exclude inter-observer variation, this study used the delineations of a single observer on CBCT. To minimize intra-observer variation, the clinical delineations on the CT scan were used as a guideline for the delineations on the CBCT. In their report on the intra-observer error of this observer (RdJ), Nijkamp et al. found maximum values of 3 mm SD for males and 2 mm for females [3].

We based plan selection on margin structures and not on the 95% isodose: in our department, this is clinical practice for image guidance for the other sites. However, perfect plan conformance will not always be possible, such as in situations with unfavorable edge-structure shapes. As a consequence, a larger margin may have been chosen than required for cases where the target volume on the CBCT lay inside the 95% isodose volume for one of the margin plans but outside the corresponding PTV.

## Conclusion

A clinically implemented adaptive plan selection strategy for rectal cancer, based on a single CT scan with variable anterior margins to the upper mesorectum, maintained coverage of the mesorectum and reduced the dose to the small bowel and bladder. For individual patients the benefit can be substantial.

## Data Availability

The datasets generated and/or analyzed during the current study are not publicly available since the participants did not consent in sharing the data with third parties.

## Abbreviations

ART: Adaptive Radiation Therapy
CBCT: Conebeam CT
CT: Computed tomography
CTV: Clinical target volume
DVH: Dose volume histogram
GTV: Gross tumor volume
Gy: Gray
IMRT: Intensity modulated radiation therapy
LCRT: Long course radiation therapy
PTV: Planning target volume
RTT: Radiation therapist
SCRT: Short course radiation therapy
VMAT: Volumetric modulated arc therapy
VxGy: Volume receiving x Gy
